# Effects of Human Umbilical Cord Mesenchymal Stem Cells on Human Trophoblast Cell Functions In Vitro

**DOI:** 10.1155/2016/9156731

**Published:** 2016-01-05

**Authors:** Yajing Huang, Yanming Wu, Xinwen Chang, Yan Li, Kai Wang, Tao Duan

**Affiliations:** ^1^Clinical and Translational Research Center, Shanghai First Maternity and Infant Hospital, Tongji University School of Medicine, Shanghai 200040, China; ^2^Department of Obstetrics, Shanghai First Maternity and Infant Hospital, Tongji University School of Medicine, Shanghai 200040, China

## Abstract

Trophoblast cell dysfunction is involved in many disorders during pregnancy such as preeclampsia and intrauterine growth restriction. Few treatments exist, however, that target improving trophoblast cell function. Human umbilical cord mesenchymal stem cells (hUCMSCs) are capable of self-renewing, can undergo multilineage differentiation, and have homing abilities; in addition, they have immunomodulatory effects and paracrine properties and thus are a prospective source for cell therapy. To identify whether hUCMSCs can regulate trophoblast cell functions, we treated trophoblast cells with hUCMSC supernatant or cocultured them with hUCMSCs. Both treatments remarkably enhanced the migration and invasion abilities of trophoblast cells and upregulated their proliferation ability. At a certain concentration, hUCMSCs also modulated hCG, PIGF, and sEndoglin levels in the trophoblast culture medium. Thus, hUCMSCs have a positive effect on trophoblast cellular functions, which may provide a new avenue for treatment of placenta-related diseases during pregnancy.

## 1. Introduction

Trophoblast cells are fundamentally involved in embryo implantation and placental development. At an early stage of pregnancy, extravillous trophoblast cells come up from the villus and penetrate into the maternal decidua and induce remodeling of the uterine spiral arteries [1–3]. Disorders during pregnancy such as preeclampsia and intrauterine growth restriction (IUGR) involve the dysfunction of trophoblast cells. For example, preeclampsia, for which the pathophysiology is not completely understood, is related to abnormal placentation. Failure of invasive trophoblasts to penetrate and convert the maternal spiral arterioles/arteries causes poor uteroplacental perfusion, which leads to a vicious cycle of cellular ischemia and hypoxia, oxidative stress, vascular endothelial injury, and the release of inflammatory factors, which eventually lead to maternal and fetal clinical symptoms [4–6]. Current treatments after diagnosis of these disorders focus mainly on improving microcirculation and blood perfusion of the organs to relieve the clinical symptoms and prevent maternal and fetal complications. However, there are seldom effective etiotropic treatments, that is, those that address the underlying cause—dysfunctional trophoblast cells.

Mesenchymal stem cells (MSCs) are self-renewal and multipotent, and, in addition, MSCs secrete a great variety of cytokines, have immunosuppressive abilities, and are not very immunogenic. Therefore, MSCs have become of interest for cell transplantation, gene therapy, and regenerative medicine during the past decades [7, 8]. Recently, Liu et al. found that injection of decidua-derived MSCs into pregnant mice with Th1-induced preeclampsia-like symptoms can alleviate hypertension and proteinuria and meanwhile prevents glomerulonephritides and facilitates placental development [9]. It has also recently been shown that chorionic plate-derived MSCs can regulate trophoblast invasion and immune responses [10]. Thus, MSCs may provide a valuable tool for remediating dysfunctional trophoblast cells, allowing them to fulfill their essential roles in placental development.

Human umbilical cord is most often treated as medical waste and is abundantly available. Compared with MSCs from other sources, human umbilical cord MSCs (hUCMSCs) have an excellent proliferation rate, a greater expansion potential, and a strong immunosuppressive capability and can be easily and noninvasively obtained from Wharton's jelly without ethical constraints, and hUCMSCs also can differentiate into osteocytes, adipocytes, chondrocytes, endothelial cells, and so on [11–13]. Therefore, hUCMSCs have been considered as seed cell candidates, and the therapeutic potential of hUCMSCs has been studied in several diseases [14]. These studies have not, however, addressed whether hUCMSCs could be beneficial to trophoblast cells.

In this study, we isolated three different human MSC lines from Wharton's jelly of umbilical cord tissue of three female neonates and examined the effects of these hUCMSCs on the cellular functions of trophoblast cells. HTR-8/SVneo is an immortalized trophoblast cell line and is usually used to study villous trophoblast cells [15–17]. We thus used the HTR-8/SVneo cell line as a model to explore the influence of hUCMSCs on proliferation, migration, invasion, and secretion functions of trophoblast cells. As MSCs can influence cells within their vicinity through both secretion of paracrine factors and cell-to-cell interactions, we analyzed the effects of hUCMSC supernatant and coculturing on these cells. To investigate whether changes were specific to hUCMSCs, normal fibroblasts (hFFs, human foreskin fibroblasts) were used as a control.

## 2. Materials and Methods

### 2.1. Reagents

The following reagents were used: Dulbecco's Modified Eagle Medium (DMEM)/F12 medium (Gibco); fetal bovine serum (FBS) (Gibco); penicillin and streptomycin solution (Gibco); trypsin (Sigma-Aldrich); the antibodies CD73-FITC, CD90-FITC, and CD105-FITC (BD Biosciences); osteogenesis differentiation kit and adipogenesis differentiation kit (Gibco); Alizarin Red S solution (Sigma-Aldrich); Oil Red O (Sigma-Aldrich); Matrigel (BD Biosciences); calcein-AM (Invitrogen); MTS (Promega); total RNA extraction kit (Tiangen); RNA reverse transcription kit (Takara); SybrGreen qPCR kit (Takara); oligo (dT) (Sangon Biotech); *β*-hCG, PIGF, and sEndoglin ELISA kits (R&D Systems).

### 2.2. Isolation of hUCMSCs

These experiments were approved by the research ethics committee at the Shanghai First Maternity and Infant Hospital. Umbilical cord samples were taken from three cesarean-delivered full-term female neonates at the Shanghai First Maternity and Infant Hospital and were immediately stored aseptically in cold saline. Umbilical cords were rinsed with phosphate-buffered saline (PBS) several times to remove blood, and the cords were dissected into short pieces. Wharton's jelly was exposed, and umbilical arteries and veins were gently removed. The chopped gel tissue (small pieces of umbilical cord Wharton's jelly) was transferred to T-75 flasks containing 10 mL medium (DMEM/F12 medium with 10% FBS and 1% penicillin and streptomycin solution) and incubated at 37°C with 5% CO_2_ and saturated humidity. The remnants of the cord fragments were removed after 7–10 days in culture, and the cultures were fed every 3 days thereafter. When cultures reached confluency, cells were trypsinized and passaged to a new flask for further expansion.

### 2.3. Detection of hUCMSC Surface Markers

After three passages, hUCMSCs were examined for surface marker expression. Cells were trypsinized and washed with and resuspend in PBS to a density of 1 × 10^6^ cells/mL. A volume of 100 *μ*L of this cell suspension was added to 1.5 mL Eppendorf tubes. Tube 1 was used as a negative control (with PBS), and the other tubes were incubated with isotype control-FITC, CD73-FITC, CD90-FITC, CD105-FITC, CD14-FITC, CD34-FITC, or CD45-FITC antibodies for 30 minutes. The cells were then analyzed using flow cytometry.

### 2.4. Osteogenic and Adipogenic Differentiation

Differentiation of hUCMSCs was performed by culturing in osteogenic or adipogenic differentiation medium in 6-well culture plates for 2-3 weeks. The medium was replaced every 2-3 days. Alizarin Red S staining was used to demonstrate the acquisition of the osteogenic phenotype. Cells were fixed with a 4% formaldehyde solution for 30 minutes, washed in PBS, and then stained with Alizarin Red S (pH 4.2) for 10 minutes. Photomicrographs were taken with a microscope (Nikon, Japan). To demonstrate adipogenic differentiation, cells were treated in parallel with Oil Red O staining instead of Alizarin Red S staining.

### 2.5. Cell Proliferation Assays

We first examined the proliferation ability of trophoblast cells in the presence of conditioned medium (different concentrations of supernatant from hUCMSCs or hFFs). 30 × 10^4^ hUCMSCs or hFFs were cultured in T-75 flasks containing 10 mL DMEM/F12 medium with 10% FBS and 1% penicillin and streptomycin solution for 24 hours; the culture medium was then collected and used after centrifugation and filtration. HTR-8/SVneo trophoblast cells in 96-well plates (2 × 10^3^ cells/well) were incubated with 0, 25, 50, or 100% hUCMSC or hFF supernatant diluted in standard medium (DMEM/F12 medium with 10% FBS and 1% penicillin and streptomycin solution) for 48 hours. Then, 10 *μ*L MTS was added to each well and incubated for 1 hour before the plates were analyzed with a microplate spectrophotometer. Additionally, we compared the proliferation ability of trophoblasts cocultured with hUCMSCs or hFFs. HTR-8/SVneo cells were placed in the lower chamber of a 6-well Transwell plate (3 *μ*m pore size, 10 × 10^4^ cells/well) and were cocultured with 0, 2.5, 5, or 10 × 10^4^ hUCMSCs or hFFs in the upper chamber. After a 48-hour incubation, the cells in lower chamber were trypsinized, and the number of HTR-8/SVneo cells was determined under the microscope.

### 2.6. Cell Migration Analysis

The migration ability of HTR-8/SVneo trophoblast cells was examined with a 24-well Transwell insert system (8 *μ*m pore size, BD Biosciences). HTR-8/SVneo cells were seeded in the upper chamber of the insert (2 × 10^4^ cells/well), which contained 300 *μ*L medium (DMEM/F12 medium with 5% FBS and 1% penicillin and streptomycin solution). The lower chambers were filled with the following: (1) 800 *μ*L condition medium (0, 25, 50, or 100% hUCMSC or hFF supernatant in standard medium) or (2) 800 *μ*L standard medium with 0, 2.5, 5, or 10 × 10^4^ hUCMSCs or hFF cells/well. After 16 hours in culture, 80 *μ*L fluorescent stain (calcein-AM) was added to each chamber and incubated for 30 minutes. The labeled cells were observed and photographed with a fluorescence microscope (Nikon, Japan).

### 2.7. Cell Invasion Analysis

The invasion ability of HTR-8/SVneo trophoblast cells was evaluated by using a 24-well Transwell insert system (8 *μ*m pore size). The upper chamber of the insert was precoated with 100 *μ*L of a 1 : 5 dilution of Matrigel in standard medium for 30 min at 37°C. HTR-8/SVneo cells (4 × 10^4^ cells/well) were then added to the upper chamber, along with 300 *μ*L of 1% FBS medium. The lower chambers were filled with the following: (1) 800 *μ*L of 0, 25, 50, or 100% hUCMSC or hFF supernatant in standard medium or (2) 0, 2.5, 5, or 10 × 10^4^ hUCMSCs or hFF cells/well along with 800 *μ*L standard medium. After 24 hours in culture, 80 *μ*L fluorescent stain was added to each chamber and incubated for 30 minutes. The labeled cells were observed and photographed with the fluorescence microscope.

### 2.8. Cell Coculture

The 6-well Transwell insert system (3 *μ*m pore size) was used for coculturing two kinds of adherent cells. HTR-8/SVneo cells (10 × 10^4^ cells/well) were added to the lower chamber, which also contained 2 mL standard medium, and hUCMSCs or hFFs were seeded in the upper chamber at a concentration of 0, 2.5, 5, or 10 × 10^4^ cells/well, with 1 mL standard medium. After 48 hours in culture, HTR-8/SVneo cells were harvested for real-time PCR and the supernatant from the lower chamber was collected for enzyme-linked immunosorbent assays (ELISAs).

### 2.9. Real-Time PCR

After coculturing for 48 hours, the upper chambers were removed, and the total RNA from HTR-8/SVneo cells was extracted using TRIzol reagent. cDNA was synthesized with the RNA reverse transcription kit and used for real-time PCR with SYBR Premix Ex Taq (Takara). PCR conditions consisted of denaturation at 95°C for 30 seconds and 40 cycles of 95°C for 15 seconds and annealing at 60°C for 20 seconds. The primer sequences were as follows: *β*-actin forward primer, 5′-CCAACCGCGAGAAGATGA-3′; *β*-actin reverse primer, 5′-CCAGAGGCGTACAGGGATAG-3′; *β*-hCG forward primer, 5′-CCAGTACCACCCCGTCATCG-3′; *β*-hCG reverse primer, 5′-CTACACGCGAAGCTC AGGTA-3′; PIGF forward primer, 5′-GCGGTACCCAAACTCATACACAATAGAC-3′; PIGF reverse primer, 5′-TTAAGCTTCCGTAGGTAAGGCTGTGGCT-3′; sEndoglin forward primer, 5′-GTCTCACTTCATGCCTCCAGCT-3′; sEndoglin reverse primer, 5′-ACTGCCTCAACATGGACAGCCT-3′.

### 2.10. ELISAs

*β*-hCG, PIGF, and sEndoglin levels in coculture experiments were measured using commercial kits as per the manufacturer's protocols, with the following modifications. Cells were labeled with the specific antibody and biotin-conjugate, followed by streptavidin-horseradish peroxidase. Within 30 minutes of labeling, the absorbance of each sample was determined with a spectrophotometer at 450 nm.

### 2.11. Statistical Analysis

All experiments were performed in triplicate. The data were analyzed for statistical significance using the SPSS software. Data are presented as the mean ± SEM. The statistical significance was tested using ANOVA and DunnT test, and comparisons between two groups were carried out with an independent *t*-test. *P* < 0.05 was considered to be statistically significant, and *P* < 0.01 was considered to be very significant.

## 3. Results

### 3.1. Isolation, Propagation, Determination, and Differentiation of hUCMSCs

**Figure 1:**
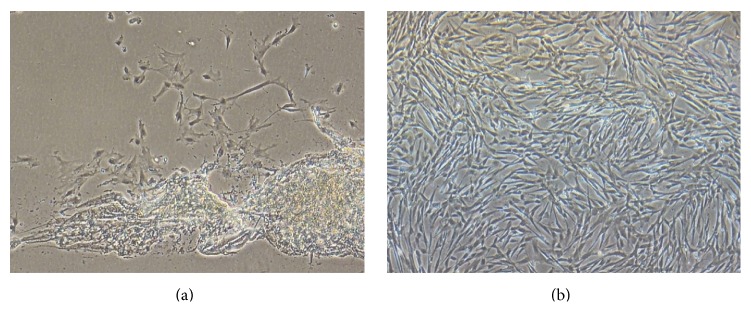
Morphological appearance of hUCMSCs. (a) The appearance of hUCMSCs on the fourth day of primary culture (100x). (b) hUCMSCs after the second passage are 90% confluent on the third day of culture (100x).

**Figure 2:**
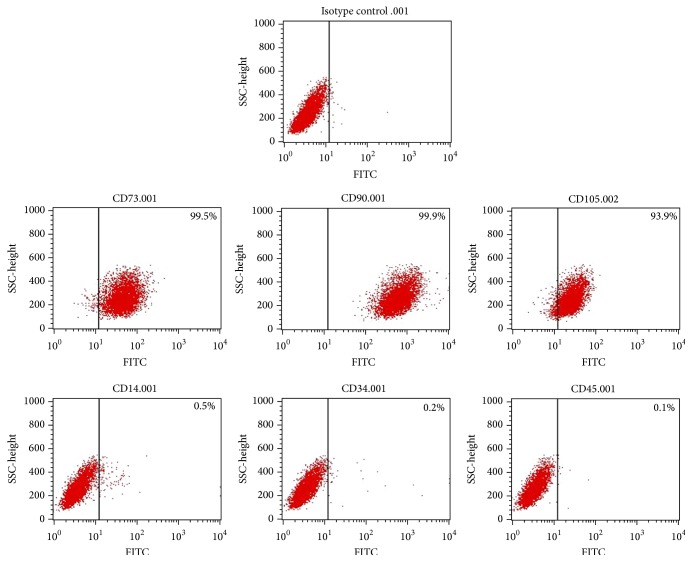
Third-passage hUCMSCs are positive for CD73, CD90, and CD105, while being negative for CD14, CD34, and CD45 as detected by flow cytometry.

**Figure 3:**
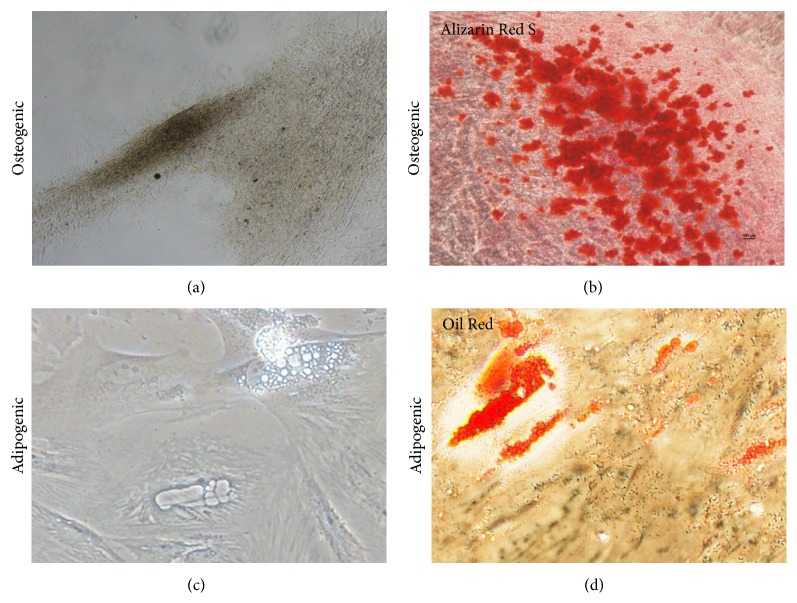
Osteogenic (a) and adipogenic (c) differentiation of hUCMSCs was assessed with Alizarin Red S (b) for osteogenesis and Oil Red O (d) for adipogenesis.

We isolated hUCMSCs successfully with the tissue block attachment method [18–20]. We saw scattered spindle-shaped cells around the tissue blocks after 4 days in culture (Figure 1(a)). These hUCMSCs reached ~80% confluency 2 weeks later and were then trypsinized and passaged at a density of 10 × 10^4^ cells/mL. After the first passage, the hUCMSCs grew very quickly and reached confluency every 3 days (Figure 1(b)). We used the cells after the third passage to detect their surface markers. These hUCMSCs were strongly positive for CD73, CD90, and CD105, while being negative for CD14, CD34, and CD45 (Figure 2), which is identical to descriptions in the literature [13, 21–24]. To test their differentiation abilities, we exposed fourth-passage hUCMSCs to osteogenic medium for 3-4 weeks. The resulting cultures were characterized by brown calcium deposition (Figure 3(a)) and osteoid formation as shown by Alizarin Red S (Figure 3(b)). Adipogenic differentiation of hUCMSCs occurred in 3 weeks. Adipocytic phenotypes of induced hUCMSCs were signaled by the appearance of tiny intracytoplasmic lipid droplets in cells; these lipid granules tended to unite (Figure 3(c)) and were stained with Oil Red O (Figure 3(d)). Those characteristics above are consistent with the minimal criteria for defining multipotent mesenchymal stem cells [25]. Meanwhile, three different hUCMSC lines in our study showed no obvious difference in their physiological effects.

### 3.2. Effect of hUCMSCs on the Proliferation Ability of HTR-8/SVneo Trophoblast Cells

**Figure 4:**
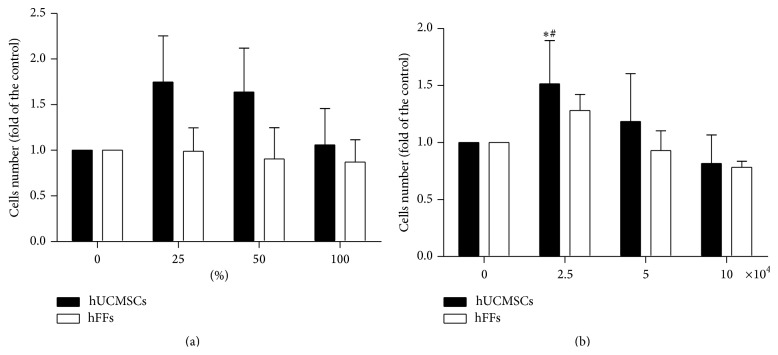
Effect of hUCMSCs and hFFs on the proliferation ability of HTR-8/SVneo cells. (a) HTR-8/SVneo cells (2 × 10^3^ cells/well) were cultured in medium containing 0, 25, 50, or 100% hUCMSC or hFF supernatant in 96-well plates for 48 hours. (b) HTR-8/SVneo cells (10 × 10^4^ cells/well) were cocultured with hUCMSCs or hFFs at a concentration of 0, 2.5, 5, or 10 × 10^4^ cells/well in 6-well Transwell plates for 48 hours. *∗* represents significant differences versus control (*P* < 0.05); # represents significant differences between hUCMSCs and hFFs at an individual dose (*P* < 0.05).

To study the influence of hUCMSCs on the proliferation ability of trophoblast cells, we used HTR-8/SVneo trophoblast cell line, one kind of human first trimester trophoblast cells, along with human foreskin fibroblasts (hFFs, donated by Bioscience Laboratory of Tongji University) as a control. We first cultured HTR-8/SVneo trophoblast cells with conditioned medium. A slight increase in HTR-8/SVneo cell proliferation was observed at 25, 50, and 100% hUCMSCs conditioned medium as compared with the medium-only control and hFFs conditioned medium; however, this increase was not statistically significant (Figure 4(a)). We also looked for effects under coculturing conditions. The proliferation of HTR-8/SVneo cells was significantly enhanced when they were cocultured with 2.5 × 10^4^ hUCMSCs/well, compared with the medium-only control and the hFFs coculture at the same density (*P* < 0.05) (Figure 4(b)).

### 3.3. Effect of hUCMSCs on the Migration Ability of HTR-8/SVneo Trophoblast Cells

**Figure 5:**
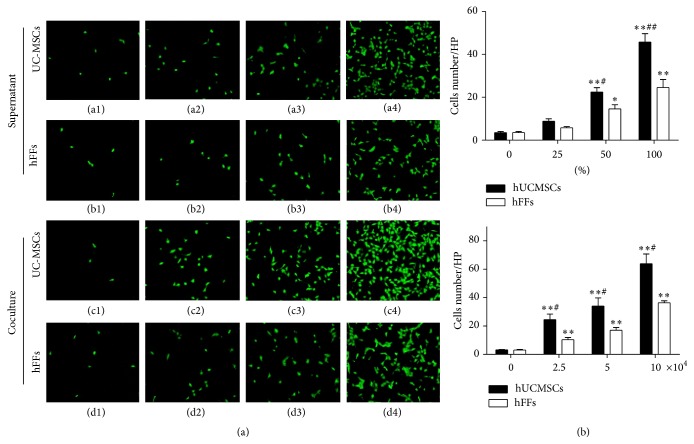
Effect of hUCMSCs and hFFs on the migration ability of HTR-8/SVneo trophoblast cells. (a) HTR-8/SVneo cells (2 × 10^4^ cells/well) were cultured in medium containing 0, 25, 50, or 100% hUCMSC (a1–a4) or hFF (b1–b4) supernatant in Transwell inserts for 16 hours or were cocultured with hUCMSCs (c1–c4) or hFFs (d1–d4) at a concentration of 0, 2.5, 5, or 10 × 10^4^ cells/well in Transwell inserts for 16 hours. Cells that had migrated were stained with calcein-AM. (b) HTR-8/SVneo cells that had migrated were counted after staining. *∗* represents significant differences versus control (*P* < 0.05); *∗∗* represents very significant differences versus control (*P* < 0.01); # represents significant differences between hUCMSCs and hFFs at an individual dose (*P* < 0.05);  ## represents very significant differences between hUCMSCs and hFFs at an individual dose (*P* < 0.01).

The migration ability of HTR-8/SVneo cells cultured with hUCMSC supernatant was significantly increased at 50 and 100% as compared with the medium-only control (*P* < 0.01) (Figures 5(a)((a1)–(a4)) and 5(b)). The migration ability of HTR-8/SVneo cells cultured with hUCMSCs supernatant was significantly higher at 50% (*P* < 0.05) and 100% (*P* < 0.01) compared with HTR-8/SVneo cells cultured with hFFs supernatant (Figures 5(a)((a1)–(a4) and (b1)–(b4)) and 5(b)). Additionally, we also carried out a similar coculturing experiment. The migration ability of HTR-8/SVneo cells cocultured with hUCMSCs was significantly upregulated at 2.5, 5, and 10 × 10^4^ cells/well compared with the medium-only control (*P* < 0.01) (Figures 5(a)((c1)–(c4)) and 5(b)). This increase was also significant compared with cells cocultured with hFFs (*P* < 0.05) (Figures 5(a)((c1)–(c4) and (d1)–(d4)) and 5(b)).

### 3.4. Effect of hUCMSCs on the Invasion Ability of HTR-8/SVneo Trophoblast Cells

**Figure 6:**
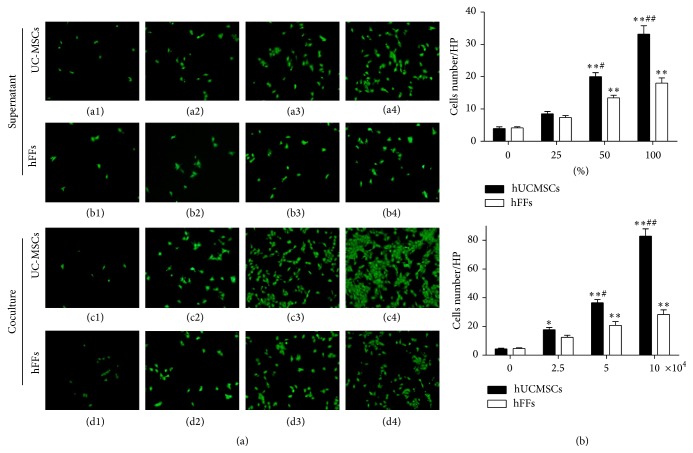
Effect of hUCMSCs and hFFs on the invasion ability of HTR-8/SVneo cells. (a) HTR-8/SVneo cells (4 × 10^4^ cells/well) were cultured in medium containing 0, 25, 50, or 100% hUCMSC (a1–a4) or hFF (b1–b4) supernatant in Transwell inserts for 24 hours or were cocultured with hUCMSCs (c1–c4) or hFFs (d1–d4) at a concentration of 0, 2.5, 5, or 10 × 10^4^ cells/well in Transwell inserts for 24 hours. Cells that had invaded were stained with calcein-AM. (b) HTR-8/SVneo cells that had invaded were counted after staining. *∗* represents significant differences versus control (*P* < 0.05); *∗∗* represents very significant differences versus control (*P* < 0.01); # represents significant differences between hUCMSCs and hFFs at an individual dose (*P* < 0.05); ## represents very significant differences between hUCMSCs and hFFs at an individual dose (*P* < 0.01).

The invasion results showed a similarity with migration. First, we compared the effects of hUCMSC and hFF supernatants. The invasive ability of HTR-8/SVneo cells cultured with hUCMSC supernatant was increased significantly at 50 and 100% compared with the medium-only control (*P* < 0.01) (Figures 6(a)((a1)–(a4)) and 6(b)). The invasive ability of HTR-8/SVneo cells cultured with hUCMSC supernatant was also significantly increased at a concentration of 50% (*P* < 0.05) and 100% (*P* < 0.01) compared with HTR-8/SVneo cells cultured with hFF supernatant (Figures 6(a)((a1)–(a4), (b1)–(b4)) and 6(b)). Additionally, in coculturing experiments, the invasive ability of HTR-8/SVneo cells was significantly enhanced when cultured with 2.5 (*P* < 0.05), 5 (*P* < 0.01), or 10 (*P* < 0.01) × 10^4^ hUCMSCs/well compared with the medium-only control (Figures 6(a)((c1)–(c4)) and 6(b)). The invasive ability of HTR-8/SVneo cells cocultured with hUCMSCs also showed an increasing trend at 5 (*P* < 0.05) and 10 (*P* < 0.01) × 10^4^ cells/well compared with HTR-8/SVneo cells cocultured with the same number of hFF cells (Figures 6(a)((c1)–(c4), (d1)–(d4)) and 6(b)).

### 3.5. Effect of hUCMSCs on the Secretion Ability of HTR-8/SVneo Trophoblast Cells

**Figure 7:**
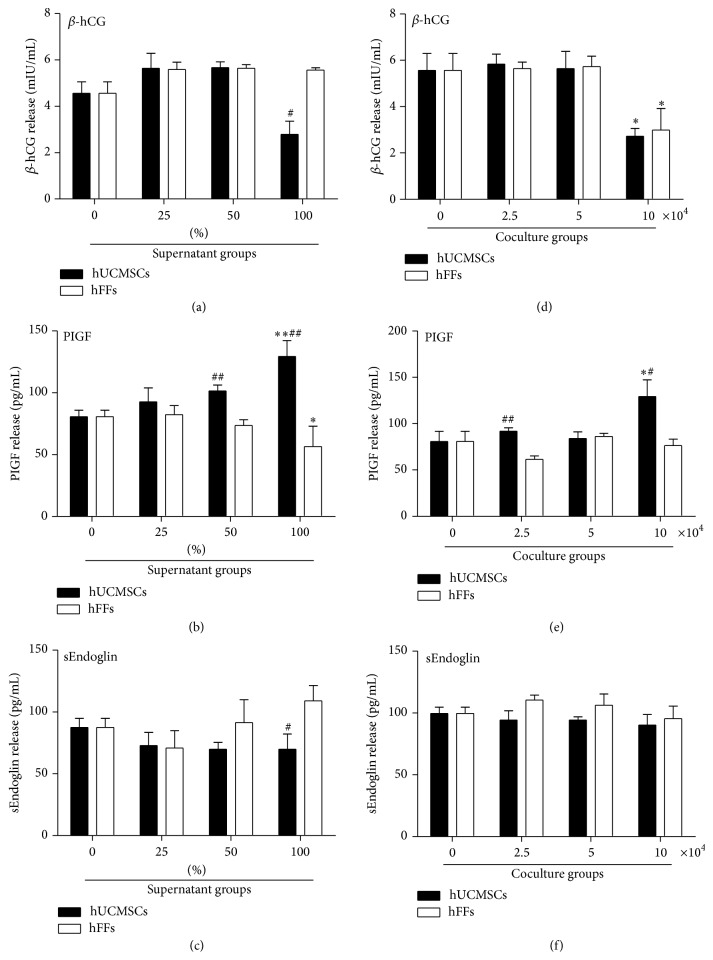
Effect of hUCMSCs and hFFs on cytokine secretion of HTR-8/SVneo cells as detected by ELISA. (a–c) HTR-8/SVneo cells (10 × 10^4^ cells/well) were cultured in 0, 25, 50, or 100% hUCMSC or hFF supernatant in medium for 48 hours. (d–f) HTR-8/SVneo cells (10 × 10^4^ cells/well) were cocultured with hUCMSCs or hFFs at a concentration of 0, 2.5, 5, or 10 × 10^4^ cells/well in 6-well Transwell plates for 48 hours. Cytokine amounts were assessed in the resulting culture medium. *∗* represents significant differences versus control (*P* < 0.05); *∗∗* represents very significant differences versus control (*P* < 0.01); # represents significant differences between hUCMSCs and hFFs at an individual dose (*P* < 0.05); ## represents very significant differences between hUCMSCs and hFFs at an individual dose (*P* < 0.01).

**Figure 8:**
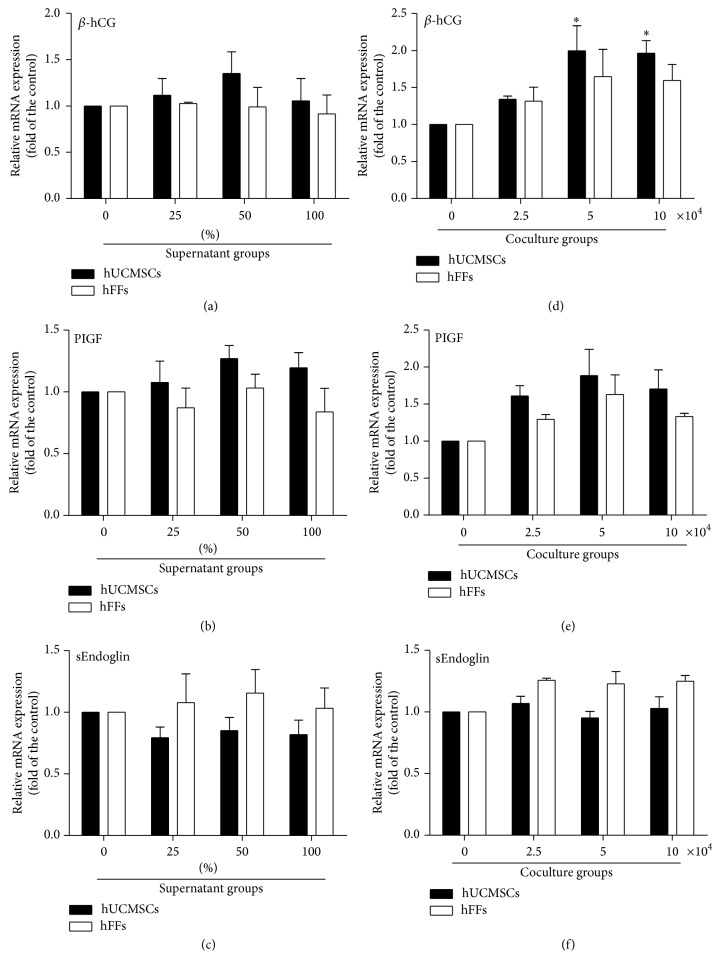
Effect of hUCMSCs and hFFs on cytokine secretion of HTR-8/SVneo cells as detected by real-time PCR. (a–c) HTR-8/SVneo cells (10 × 10^4^ cells/well) were cultured in 0, 25, 50, or 100% hUCMSC or hFF supernatant in medium for 48 hours. (d–f) HTR-8/SVneo cells (10 × 10^4^ cells/well) were cocultured with hUCMSCs or hFFs at a concentration of 0, 2.5, 5, or 10 × 10^4^ cells/well in 6-well Transwell plates for 48 hours. Cytokine amounts were assessed in the resulting culture medium. *∗* represents significant differences versus control (*P* < 0.05); *∗∗* represents very significant differences versus control (*P* < 0.01); # represents significant differences between hUCMSCs and hFFs at an individual dose (*P* < 0.05).

We detected a series of cytokines in the coculture medium in the preliminary experiments (data not shown) and ultimately chose *β*-hCG, PIGF, and sEndoglin for the evaluation of hUCMSC effects on the secretion function of trophoblasts. We added 0, 25, 50, or 100% hUCMSC or hFF supernatant in medium to 6-well plates seeded with 10 × 10^4^ HTR-8/SVneo cells/well. After 48 hours in culture, we collected the supernatant for ELISA detection and extracted the total RNA from the cells for real-time PCR detection. hUCMSC conditioned medium had no effect on secreted *β*-hCG or mRNA of *β*-hCG, although at 100% there was a significant reduction in the level compared with hFF conditioned medium (Figures 7(a) and 8(a)). The concentration of PIGF was significantly upregulated with 50 and 100% hUCMSC supernatant compared with hFF supernatant and, for the 100% supernatant only, with the medium-only control (Figure 7(b)). The mRNA level of PIGF showed a slightly rising trend, but no significant change (Figure 8(b)). The concentration of sEndoglin was significantly downregulated with 100% hUCMSC supernatant compared with 100% hFF supernatant, although no effects were seen as compared with the medium-only control (Figure 7(c)). The real-time result also showed a slightly downward trend, but no significant change (Figure 8(c)).

In addition, we carried out coculturing experiments. HTR-8/SVneo cells (10 × 10^4^ cells/well) were added to the lower chamber of a 6-well Transwell insert system, and hUCMSCs or hFFs were seeded in the upper chamber at a concentration of 0, 2.5, 5, or 10 × 10^4^ cells/well. After 48 hours in culture, samples were collected and assessed as described above. The concentration of *β*-hCG showed no significant change in the presence of 2.5 or 5 × 10^4^ cells/well, but it decreased in the presence of 10 × 10^4^ hUCMSC or hFF cells/well compared with the medium-only control (Figure 7(d)). However, the mRNA level of *β*-hCG significantly increased at 5 and 10 × 10^4^ cells/well, comparing with the medium-only control (Figure 8(d)). The concentration of PIGF was significantly upregulated by coculturing with 2.5 or 10 × 10^4^ hUCMSCs/well compared with hFFs and, for the higher concentration, with the medium-only control (Figure 7(e)). The mRNA level of PIGF showed a slightly rising trend, but no significant change (Figure 8(e)). There was no significant change in the concentration of sEndoglin, as well as the mRNA level of sEndoglin in cocultures with hUCMSCs compared with the medium-only control or hFFs (Figures 7(f) and 8(f)).

## 4. Discussion

As the major resident cells at the fetal-maternal interface, trophoblast cells play considerable roles in embryonic development during early pregnancy. hUCMSCs are an excellent source of mesenchymal stem cells and have been well studied in the context of different fields and diseases. Here, we examined the effects of hUCMSCs on the proliferation, migration, invasion, and secretion functions of trophoblast cells. hUCMSC supernatant or coculturing with hUCMSCs can facilitate trophoblast cell functions at certain concentrations in contrast with medium-only controls or hFFs.

The proliferation of trophoblast cells forms the basis of embryo implantation [26, 27]. The proliferation ability of HTR-8/SVneo trophoblast cells was significantly elevated in cocultures with 2.5*∗*10^4^ cells/well hUCMSCs, but this effect was not in the presence of hUCMSC conditioned medium. The gradual decline in cell number as the concentration of hUCMSC supernatant increased (Figure 4) may have resulted from a depletion of nutrients in the medium and the resultant reduction in the secretion of mediating factors by hUCMSCs, and the specific mechanisms need more intensive studies.

Migration and invasion are the most important functions of trophoblast cells, and many studies have confirmed that inadequate trophoblast invasion is associated with complications during pregnancy such as preeclampsia [1, 28–31]. hUCMSCs supernatant as well as coculturing with hUCMSCs had a significant effect on the migration and invasion abilities of HTR-8/SVneo cells. In contrast, hFF cells had a positive albeit significant smaller influence on the migration and invasion abilities of these trophoblast cells. Choi recently demonstrated that chorionic plate-derived mesenchymal stem cells that are cocultured with trophoblasts can also promote the invasion ability of trophoblast cells, which is consistent with our findings, and the activity of MMP-2 in the trophoblast cells was significantly increased [10]. Studies found that matrix metalloproteinases (MMPs) are highly expressed and synthesized in human MSCs, and the inflammatory cytokines such as TNF-*α* can upregulate MMP-2, MT-1 MMP, and MMP-9 production in these cells [32–35]. However, the complex mechanisms were still not very clear.

Cytokines and hormones are also involved in regulatory mechanisms of embryo implantation. hCG, a dominant hormone during pregnancy, has many important functions, including the promotion of implantation and decidualization [36], angiogenesis, facilitation of trophoblastic differentiation [37], production of progesterone [38], and regulation of immune cells [39], all of which are central to the establishment of the fetomaternal interface. In addition, reduced hCG secretion in the first trimester of pregnancy may lead to deficient placentation and a higher risk of disorders during pregnancy [40, 41]. Pizarro recently reported that hCG can increase MMP-2 secretion and decrease TIMP-1 secretion by human endometrial stromal cells and hence induce changes in remodeling of the surrounding extracellular matrix and promote HTR-8/SVneo trophoblast cell invasion in vitro [42]. However, the ELISA test did not show significantly the upregulation effect, while real-time PCR test showed that coculturing with 5 or 10*∗*10^4^ cells/well hUCMSC can significantly increase the mRNA level of hCG, comparing with medium-only control. So, no definite conclusion could be made from the above data, and the reasons for the decrease of hCG (Figure 5) (due to differentiation or cell death?) need more researches.

In addition, PIGF is a secreted proangiogenic protein that belongs to the VEGF family. PIGF participates in the development of the intrauterine vascular network, trophoblast invasion, and inflammatory processes [43]. Decreased maternal serum levels of PIGF during early gestation correlate with an increasing risk of abnormal pregnancy and, specifically, early-onset preeclampsia [44]. hUCMSCs could upregulate the concentration of PIGF in medium in both supernatant group and coculture group, as compared with the medium-only control or hFF cells, suggesting that hUCMSCs have a beneficial effect on trophoblast cells through facilitation of PIGF synthesis and release, or through an increase in PIGF secretion (hUCMSCs also secrete PIGF as many studies confirmed).

Alterations in circulating antiangiogenic protein levels are also involved in the pathogenesis of many disorders during pregnancy. Endoglin is expressed in the syncytiotrophoblast throughout pregnancy. Endoglin modulates responses to several TGF-*β* superfamily ligands and is essential for the negative regulation by TGF-*β* isoforms 1 and 3 during extravillous trophoblast differentiation. Endoglin also binds endothelial nitric oxide synthase and regulates its activation and vasomotor tone [45, 46]. Mano also found that the loss of sEndoglin can promote the invasion ability of extravillous trophoblasts [47]. The data showed that 100% hUCMSC supernatant could significantly downregulate the concentration of sEndoglin comparing with 100% hFF supernatant, and the mRNA level of sEndoglin showed a slightly downward trend, but no significant change, which suggests a beneficial effect of hUCMSCs on HTR-8/SVneo trophoblasts.

In conclusion, hUCMSCs at certain concentrations in vitro, in the form of supernatants or cocultures, can be beneficial for the proliferation, migration, invasion, and secretion function of HTR-8/SVneo trophoblast cells, which provides new insights into potential therapeutic or preventive approaches for placenta-related disorders during pregnancy. Since symptoms of pregnancy disorders like preeclampsia do not occur typically until after 20 weeks, by which time trophoblast invasion (or its failure) is long completed (usually done by 8–10 weeks of gestation), even if hUCMSCs alter HTR-8/SVneo biology, it is not clear how this could be translated into clinical practice. One possible application might be that pregnant women who have risk factors of pregnancy disorders could receive hUCMSCs treatment for prevention. On the other hand, HTR-8/SVneo represents only one cell type, and our analysis of this cell line occurred in isolation from its original microenvironment and tissue architecture. Whether hUCMSCs would have similar effects on other kinds of cells in the placenta or even in vivo is unknown. Meanwhile, we only isolate three hUCMSC lines in our study; although they showed no obvious difference in their physiological effects, still the results were not representative of the entire population, and studies containing more hUCMSC lines are needed in future. Besides, all of the three hUCMSC lines are isolated from female neonates, and as the NIH and APS suggest that sex difference maybe influences physiology, further study should investigate whether the sex of hUCMSC lines influences physiological functions to support trophoblast cell migration and invasiveness. We also do not know the mechanism responsible for these effects. We detect miR-181a in hUCMSCs, which may play a role in the mechanism, but did not get a satisfactory result yet. However, the interaction of hUCMSCs and trophoblasts still opens a new gate for this idea, and more researches will be carried out in future.
